# Gut Microbiota–microRNA Interactions and Obesity Pathophysiology: A Systematic Review of Integrated Studies

**DOI:** 10.3390/ijms252312836

**Published:** 2024-11-29

**Authors:** Hushyar Azari, Megan George, Kembra Albracht-Schulte

**Affiliations:** Department of Kinesiology and Sport Management and Obesity Research Institute, Texas Tech University, Lubbock, TX 79409, USA; hazari@ttu.edu (H.A.); megangeo@ttu.edu (M.G.)

**Keywords:** adipose tissue, gut microbiota, micro-RNA, obesity

## Abstract

Obesity is the fifth leading cause of death globally and its comorbidities put a high burden on societies and cause disability. In this review, we aim to summarize the interactions and crosstalk between gut microbiota and micro-RNA (miRNA) in obesity. We searched for the relevant literature through PubMed, Web of Science, Scopus, and Science Direct. The study design is registered in the international prospective register of systematic reviews (Prospero). According to the inclusion criteria, eight studies were eligible for assessment (two studies including human subjects and six studies including animal subjects). We report that the interactions of miRNA and gut microbiota in the context of obesity are diverse and in some cases tissue specific. However, the interactions mediate obesity-associated pathways including the inflammatory response, oxidative stress, insulin signaling, gut permeability, and lipogenesis. To mention the most meaningful results, the expression of adipose tissue miRNA-378a-3p/5p was associated with *Bifidobacterium* and *Akkermansia* abundance, the expression of hepatic miRNA-34a was related to the *Firmicutes* phylum, and the expression of miRNA-122-5p and miRNA-375 was associated with the *Bacteroides* genus. miRNA-microbiota-associated pathological pathways seem to provide an intricate, but promising field for future research directed toward the treatment of obesity and its comorbidities.

## 1. Introduction

Increasing obesity prevalence across all age groups has become a major global health challenge, since it is a major risk factor for noncommunicable diseases (NCDs), including type 2 diabetes mellitus (T2DM), cardiovascular diseases, fatty liver, and cancer [1,2,3,4]. It is estimated that about one billion people in the world will suffer from obesity in 2030, and the consequent comorbidities will continue to be a substantial burden on the world economy and healthcare systems [4,5,6,7]. According to the Centers for Disease Control and Prevention (CDC), obesity costs the United States about 173 billion dollars per year [8].

Obesity pathogenesis is multifactorial [9,10,11] and is the result of an interplay among lifestyle, environmental factors, genetics, epigenetics—including miRNA and DNA methylation—, endocrine system disruptions, and alterations in the gastrointestinal (gut) microbiome and related metabolites [12,13,14]. Various tissues and organs contribute to and are impacted by obesity pathophysiology, including adipose tissue—specifically visceral adipose tissue (VAT) [9,15]– and the liver [16], both of which are key contributors to obesity-associated systemic inflammation and insulin resistance [17]. Recent obesity research is highly focused on the complex contributions of gut microbiota and epigenetic factors, given the plethora of genes directly or indirectly related to obesity pathophysiology. Changes in bacterial composition and their metabolic products, as seen in obesity, alter specific miRNA in pathways associated with metabolic dysfunction [18]. Concurrently, miRNA from various tissues directly regulate gut microbiota [19,20]. Indeed, the cecal miRNA signature is dependent on the presence of endogenous microbiota in mice [21]. These complex connections and interactions provide opportunities to study novel pathologic pathways regarding obesity and its comorbidities [22,23].

### 1.1. Gut Microbiota Interactions with Adipose Tissue and Liver

The human gut is home to more than 100 trillion microorganisms, including bacteria, fungi, viruses, and archaea, with the colon possessing the highest density [24,25]. Bacteria in the gut have numerous functions, including helping in digestion, the synthesis of different vitamins and other active metabolites, as well as the modulation of the immune response [24,26]. Gut microbiota have been shown to extensively affect different metabolic pathways and immune responses and thus are involved in many disorders and diseases such as obesity [27]. It is important to mention that even minor perturbations in gut microbiota composition during early life, which are shaped by different factors such as delivery mode, pregnancy age, feeding practices, antibiotic exposure, and ecological factors, have a remarkable influence on the core microbiome and can be related to developing obesity in adulthood [28,29].

The majority of human gut microbiota can be classified under one of four phyla: *Proteobacteria*, *Bacteroidetes*, *Actinobacteria*, and *Firmicutes* [30]. An equilibrium among different gut microbiota populations, which is characterized as rich, even, and diverse, is extremely important for maintaining the health of the host. Healthy or “good” gut microbiota produce short chain fatty acids (SCFAs)—butyrate, acetate, propionate, valerate, and formate—and vitamins, regulate bowel movements, and aid in the recovery of the equilibrium state [31]. SCFAs have diverse biological effects, including maintaining the diversity and integrity of gut microbiota composition, maintaining normal intestinal acid/base balance, preventing the growth of pathogenic bacteria, improving insulin sensitivity and modulating energy metabolism, providing energy for colonocytes, and strengthening gut barrier integrity [32,33].

Changes in the diversity, richness, evenness, and ratios of specific bacterial phyla and their metabolites results in dysbiosis and contribute to a variety of pathologies [34,35] via changes in the concentrations of vitamins and SCFAs, changes in immune responses, and the growth of pathogenic bacteria [30]. Another way that gut microbiota can drive obesity is via lipopolysaccharide (LPS), which is a critical mediator of the negative effects associated with the overgrowth of pathogenic bacteria and dysbiosis [36]. LPS is a component of gram-negative bacteria that, if translocated through intestinal tight junctions, causes endotoxemia and contributes to adipocyte hyperplasia and the recruitment of macrophages to VAT [36,37]. Systemic endotoxemia not only drives inflammation in WAT, but also exerts its effects on different obesity-related tissues such as the liver, brain, and pancreas, exacerbating metabolic dysfunction and resulting in the progression of obesity [38,39].

A bidirectional relationship, in terms of function and anatomy, exists between gut microbiota and the liver due to their proximity via portal circulation [40]. Gut epithelial cells and their tight junctions, immunoglobulins, mucin, immune cells, and commensal bacteria are all involved in the complex regulation of the microbiota, metabolites, and liver microenvironment [40]. Disruptions in gut barrier integrity direct pathogens and their associated molecules toward liver tissue, leading to toll-like receptor (TLR) activation that contributes to hepatic inflammation [41]. Likewise, liver-derived factors, such as bile acids and hepatokines, are essential elements that can modulate gut microbiota composition and also affect the function of intestinal integrity, thus impacting overall gut health [42]. Primary bile acids, including cholic acid (CA) and chenodeoxycholic acid (CDCA), are metabolized to secondary bile acids, including lithocholic acid (LCA) and deoxycholic acid (DCA), by the gut microbiota and can affect tissues, including WAT, pancreas, and muscle tissues, and play a role in energy homeostasis, immunity, and intestinal integrity [43,44,45]. Hepatokines regulate immune responses inside the gut, further refining its microbial environment [42,46]. Indeed, gut microbiota dysbiosis is an important factor involved in the pathology of different liver diseases such as alcoholic liver disease, metabolic dysfunction-associated steatotic liver disease (MASLD), viral hepatitis, and hepatocellular carcinoma [40,47].

Excessive amounts of fat accumulation in WAT leads to an increased number of pro-inflammatory macrophages and the secretion of several cytokines (adipokines) that affect several physiological processes [48,49]. Additionally, increases in bacterial-associated products, such as LPS, in the circulation can eventually cause increased WAT inflammation and obesity [48]. Furthermore, brown adipose tissue (BAT), which is known for its role in thermogenesis and energy expenditure, contrasts with WAT and is also recognized for its potential interactions with the gut microbiota [50]. Confounding studies have shown both an increase in the prevalence of beige adipocytes as well as a decrease in WAT browning and impaired thermogenic activity of BAT following antibiotic-induced microbiota depletion [49,50,51]. This highlights the complex connection between the composition of gut microbiota and adipose tissue regulation and suggests that gut status may directly influence the metabolic functions of both WAT and BAT [52].

### 1.2. microRNA Interactions with Adipose Tissue, Liver, and Intestines

MicroRNAs (miRNA) are small, non-coding RNAs involved in post-transcriptional gene expression [53,54] and the prevention of messenger RNA translation [55,56,57]. These molecules can be found inside the cell and regulate the expression of genes in an endogenous way or by acting exogenously in the shape of exosomes [58,59]. These molecules are widely expressed in different tissues of the body and can be measured in blood, fecal samples, cerebrospinal fluid, saliva, and various tissues such as liver tissue, adipose tissue, and the gastrointestinal tract [56]. Since these molecules can be easily measured in the blood, they can be used as potential biomarkers for various diseases/disorders, including obesity, stroke, diabetes, and cardiovascular diseases [56,60]. Various miRNA families are associated with obesity, such as the miRNA-221, miRNA-223, miRNA-122, miRNA-192, miRNA-130, and miRNA-378 families [22,61], which are all highlighted in recent reviews [62,63,64]. The secretion of miRNAs from various body tissues, including WAT, the liver, and the intestines, presents extensive research opportunities to explore their preventative and therapeutic potential in managing obesity and its associated metabolic disorders [13,65].

miRNAs are involved in the regulation of WAT metabolism and have been associated with insulin signaling, mitochondrial biogenesis, and lipid profile [63,66]. Specific miRNAs, including miRNA-143, miRNA-130, and miRNA-21, are key miRNAs associated with different aspects of obesity pathophysiology such as WAT metabolism, WAT insulin resistance, and inflammation [62,67,68]. The majority of circulating miRNA originate from WAT, and when adipocytes become dysfunctional due to obesity, these miRNA reach other organs, including the liver and large intestine [69] and exacerbate obesity complications via alterations in gene expression related to pathways involved in metabolism and inflammation [63,69,70].

miRNAs are also involved in lipid metabolism, inflammation, and insulin signaling in the liver [71] and are altered with obesity progression [65]. As the degree of steatosis and inflammation increases in the liver, the expression level of specific miRNA is altered and is specific to the stage of liver disease [72]. For example, both miRNA-34a and miRNA-122 are increased in individuals with MASLD; however, miRNA-34a increases with disease progression and miRNA-122 decreases with disease progression [72]. miRNA-34a is associated with the inhibition of lipid metabolism and miRNA-122 is related to enhanced lipid metabolism [73,74].

About 453 families of miRNAs have been identified in both the small and large intestines, and these miRNAs contribute to homeostasis in the intestines [75]. miRNAs can interact with diverse elements present in the intestines, such as immune system mediators, intestinal epithelial cells, and intestinal microbiota, and through these interactions can promote or prevent diseases [19,76]. As mentioned previously, an important function of intestinal epithelial cells is providing gut barrier integrity via tight junction proteins [75,77]. Intestinal miRNA-122a functions as a key regulator of these tight junctions. Specifically, in inflammatory status, increased TNF-α causes the higher expression of intestinal miRNA-122 which degrades claudin mRNA, a tight junction protein [75,78], thus connecting miRNA-122 to intestinal permeability and dysbiosis etiology.

The bidirectional relationship that exists between gut microbiota and miRNAs is characterized by miRNAs targeting bacterial genes, which then can modulate microbiota composition through their post-transcriptional effects. Moreover, gut microbiota also influence gene expression and miRNA generation via bacterial metabolites such SCFAs and LPS [79]. The role of microRNA in the regulation of gut microbiota and the consequent health benefits or complications is measurable. Since probiotics alter miRNA expression [79], significant associations may exist between obesity and related diseases and gut microbiota–miRNA interactions [20,79,80]. Therefore, we aim to elucidate the associations and interactions of gut microbiota and miRNA in the context of obesity pathophysiology and its comorbidities.

## 2. Methods

### 2.1. Search Strategy and Registration

A systematic search was performed using Mesh terms and non-MESH terms using online databases, including Science Direct, Web of Science, PubMed, and Scopus. The search strategy consisted of [microRNA] or [miRNA] and [gut microbiota] or [gut microbiome] and [obesity] or [overweight]. We also conducted a manual search of references from the selected articles. This review is registered in the International Prospective Register of Systematic Reviews (PROSPERO) with the registration ID: CRD42023401387.

### 2.2. Eligibility Criteria

**Figure 1:**
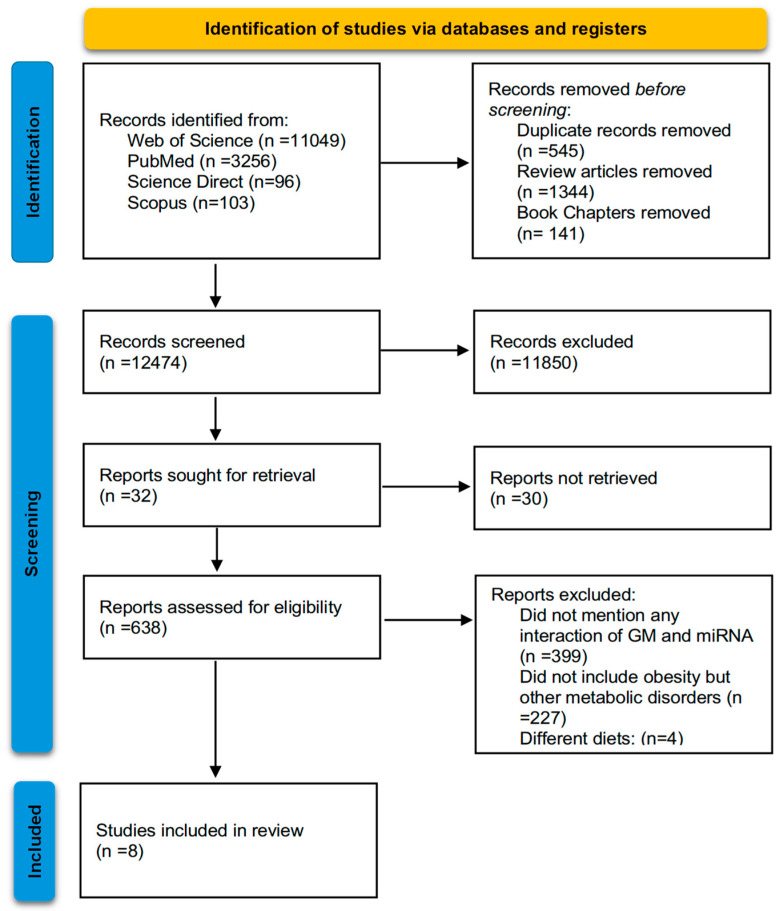
The PRISMA flow diagram of the systematic review.

Studies assessing the association and interactions of miRNA and gut microbiota in obesity were selected. Any English-written publication after 2016 that investigated the association and interaction of miRNA and gut microbiota as related to obesity pathophysiology in (a) animals or (b) humans and as a (i) case–control study or (ii) clinical trial was collected. This process took place in two phases: (1) title and abstract assessment; and (2) full text assessment. This selection process was conducted by all members of the team and all studies were investigated. In cases of indecisiveness, article eligibility was discussed among the members until a final decision was made by the direct supervision of the corresponding author. In accordance with PRISMA, a flow chart showing article selection and exclusion is shown in Figure 1.

### 2.3. Data Collection

Data regarding bacterial species, study populations, the miRNA types assessed, changes in gut microbiota, and miRNA from baseline and different pathophysiologic pathways associated with the alterations were organized into tables.

## 3. Results

### 3.1. Main Characteristics of the Included Studies

Overall, there were a minimal number of studies (eight articles total) that were in line with our criteria and assessed the interactions of both gut microbiota and miRNA in relation to obesity pathophysiology. Of these, six [18,81,82,83,84,85] studies were conducted using animal subjects (Table 1), and two [86,87] were conducted using human subjects (Table 2). Two studies were conducted in the USA [18,88], four in Asia [83,84,85,87] and two in Europe [81,82].

### 3.2. miRNA–Gut Microbiota Dynamics

**Figure 2:**
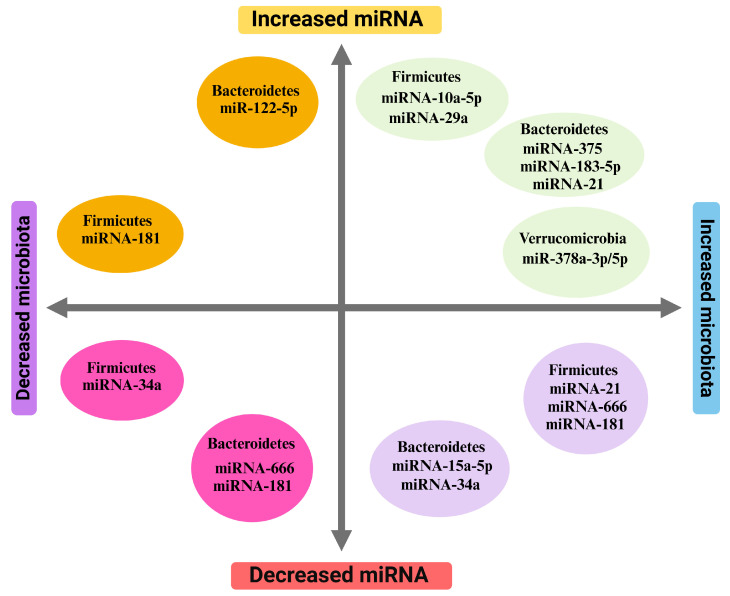
Associations between specific micro-RNAs and gut microbiota phyla according to Table 1 and Table 2 (distances from x- and y-axes do not show greater increase or decrease).

The interactions and interplay of gut microbiota and miRNA are very intricate, with some consistency among interactions across the various tissues and in the animal and human studies explored in this review. Gut microbiota–miRNA associations are shown in Figure 2.

The *Firmicutes* phylum is positively correlated with hepatic miRNA 34a, while being negatively correlated with hepatic miRNA-666 [81,82]. Similarly, the *Bacteroidetes* phylum is positively correlated with hepatic miRNA 122 and circulating miRNA 375 [82,86]. Additionally, the *Akkermansia* from *Verrucomicrobiota* phylum displayed positive correlation with fecal miRNA-42f5-3p and WAT miRNA-378a [83]. The *Actinomycetota* phylum, and specifically *Bifidobacterium* genus, is positively associated with adipose tissue miRNA-378a [83]. It is worth noting that the miRNA-378a family in WAT and liver demonstrated associations with SCFA-producing bacteria such as *Lactobacillus* genus [83].

While there are additional studies which have investigated microbiota–miRNA interactions, we excluded some of them because of the variations in their dietary interventions that could confound the results. For instance, in a study by Liu et al., it was shown that increased expression of miRNA-1226-5p was associated with increased abundance of *Proteobacteria* in stool samples [20], and in another study by Deng et al., higher *Bifidobacterium* levels were linked to increased GI miRNA-5112, while a decrease in *Bacteroidetes* was associated with the downregulation of liver miRNA 134-5p and miRNA 543-3p [89]. Additional studies in humans have documented associations between increased *Verrucomicrobiota* and *Firmicutes* with elevated miRNA-425-3p and miRNA-638 expression [90].

### 3.3. Findings of Animal Studies

#### 3.3.1. Hepatic miRNA and Gut Microbiota

High-fat feeding in mice results in complicated changes in gut microbiome composition and the expression profile of miRNA. This diet results in an increased abundance of the *Firmicutes* phylum, which is associated with elevated hepatic levels of miRNA-34a and miRNA-29a [82,85]. Conversely, higher hepatic expression of miRNA-666 and miRNA-21 correlates with a decline in *Firmicutes* and a rise in *B. acidifaciens* in the gut [81]. This diet also causes a reduction in *Bacteroidetes* alongside decreased miRNA-122 levels [82].

#### 3.3.2. WAT miRNA and Gut Microbiota

An increase in *Clostridium* and other indole-producing bacteria is associated with the overexpression of WAT miRNA-181 [18]. Additionally, increased *Akkermansia muciniphila*, *Lactobacillus*, *Bifidobacterium*, and *clostridium* is linked with an increase in WAT miRNA-378a [83].

#### 3.3.3. Microbiota and Externally Provided miRNA

In a study by Guo et al., increased levels of miRNA-10a-5p in the GI tract promoted the expansion of *Lachnospiraceae* and its metabolite butyrate [84].

### 3.4. Findings of Human Studies

There was a significant positive relationship between *B. eggerthi* and circulating miRNA-375 [86]. Higher fecal miRNA-425-3p expression was associated with a greater abundance of *A. muciniphila* and *Roseburia* sp., and higher fecal miRNA-638 correlated with a lower abundance of *A. colihominis* [90]. In addition, circulatory miRNA-122-5p is negatively related with *Bacteroides* [87].

## 4. Discussion

Obesity has been declared a worldwide epidemic by the World Health Organization (WHO) since 1997, and its increasing prevalence has created additional health and economic challenges [36,91,92]. One remarkable aspect of obesity pathophysiology is the effects of different gut microbiota and specific miRNAs associated with this condition as well as the interaction/crosstalk between the two that may contribute to the complexity of the disease. For example, *B. eggerthii* (*Bacteroidetes phyla*) is elevated in obesity and positively correlated with body fat percentage in human subjects [86,93,94] and with the elevated expression of circulating miRNA-130b-3p, miRNA-185-5p, miRNA-183-5p, and miRNA-21-5p [86]. miRNA-183-5p, in particular, is related to increased adipogenesis according to in vitro studies using 3T3-L1 adipocytes [86,95,96]. Therefore, gut microbiota–miRNA crosstalk is highly relevant and is an emerging and understudied component of obesity pathophysiology [91,97]. Most studies in this review reported gut microbiota–miRNA interactions related to the expression of genes in interconnected pathways and processes including inflammation, oxidative stress, gut barrier function, gut microbiota-derived metabolite production, endocrine signaling, and insulin signaling.

Chronic low-grade inflammation, which is in part characterized by the excessive production of pro-inflammatory cytokines, such as TNF-α, IL-1β, IL-12, and IL-6 from expanded WAT, is a key driver of the metabolic dysfunction associated with obesity [98]. Additionally, gut barrier integrity, or intestinal permeability, can cause or aggravate inflammation and metabolic disorders if disturbed [99]. Certainly, the complex interactions between gut microbiota and miRNA contribute to obesity-associated inflammation [86]. It was demonstrated using Dicer1 knock-out mice that miRNAs had essential functional roles, such as epithelial cell proliferation, nutrient absorption, and cell migration, and that defects in miRNA biogenesis were responsible for impaired intestinal barrier function [100]. Although not linked to a specific bacteria, miRNA-10a is downregulated in inflamed intestinal cells and is associated with increased levels of its target IL-12 (IL-23p40), a pro-inflammatory cytokine that activates M1 macrophage polarization in the WAT of C57BL/6 mice [101,102,103]. Furthermore, ZO-1, a marker of gut barrier strength, is significantly expressed in the gastrointestinal villus of miRNA-29a-overexpressed mice [85]. Moreover, inhibiting the expression of intestinal miRNA 21-5p decreases the expression of tight junction proteins, occludin and claudin, thereby increasing gut permeability in BALB/c mice [104]. Therefore, miRNAs are key molecules in the maintenance of gut barrier integrity; thus, they are unsurprisingly associated with changes in specific bacteria and inflammation [103]. For example, Liu et al. showed that fecal miRNA-30d was associated with an increase in *A. muciniphila* (*Verrucomicrobiota phyla*) in C57BL/6J mice, which has been associated with improved gut integrity by increasing the expression of occludin [105,106]. As a mechanism of gut microbiota–miRNA interactions, Liu et al. reported that miRNA-30d can be internalized by *A. muciniphila* and affect bacterial gene expression [105]. However, further research is warranted to elucidate the complexity of these mechanisms.

Moreover, key bacterial phyla from the reviewed studies include *Firmicutes*, *Bacteroidetes*, *Verrucomicrobiota*, and *Thermodesulfobacteriota*. A decreased abundance of *Firmicutes*, a decreased *Firmicutes* to *Bacteroidetes* ratio (F/B), and a lower expression of IL-6 concentration after overexpression of hepatic miRNA-29a (anti-inflammatory) may be relevant to obesity and MASLD in C57BL/6 mice [85]. Additionally, contrasting findings exist regarding the association between *Firmicutes* and miRNA-21, and miRNA-34a with liver triglycerides [81], with upregulated miRNA-34a being associated with fatty liver disease in both animals and humans [82,107,108,109]. Hepatic miRNA-122 has been associated with decreased *Bacteroides* (*Bacteroidetes phyla*) in human subjects, which may be an interaction driving hepatic lipogenesis since in vitro miRNA silencing studies have demonstrated changes in genes related to fatty acid synthesis [110]. However, there still is no consensus that the ratio of F/B is consistently higher in obese individuals due to differences in methodological approaches, different dietary interventions, and differences in participant characteristics [85,103,111].

Additionally, *A. muciniphila* (*Verrucomicrobiota phyla*), which is positively associated with WAT miR-378a in mice [83], has been linked to a reduction in pro-inflammatory cytokines including interlukin 2 (IL-2); interferon-gamma(IFN-γ), monocyte chemoattractant protein-1(MCP-1), and apolipoprotein B-48 (apoB48) on chylomicrons; and apolipoprotein B100 (apoB100) on low density lipoprotein (LDL) in mice studies [106]. In contrast, a higher abundance of *Bacteroides* (*Bacteroidetes phyla*), *Parabacteroides* (*Bacteroidetes phyla*), and *Bilophila* (*Proteobacteria phyla*) is positively correlated with hepatic miRNA-5112 regulators of pro-inflammatory genes, including leptin receptor (*Lepr*), oncostatin M receptor (*Osmr*), protein kinase B (*Akt1*), the suppressor of cytokine signaling 3 (*Socs3*), galectin-9 (*Lgals9*), and histone deacetylase 1 (*Hdac1*) in C57BL/6J mice [89]. Furthermore, Deng et al. used a known healthy microbiota population as a standard compared to the dysbiotic microbiota population in their study to assess the miRNAs which had altered expression in those two microenvironments [89], and it was shown that a higher abundance of *Bacteroidetes* and a lower abundance of *Bifidobacterium* (*Actinobacteria phyla*) in the dysbiotic microbiota population were correlated with pro-inflammatory and anti-inflammatory genes, respectively, and also their miRNA regulators, including miRNA-5112 and miRNA-342-3p in the liver [89].

Insulin resistance is a hallmark of obesity in which the metabolic response to insulin is impaired [112]. miRNA-181a and miRNA-181b are more prevalent in the WAT of obese humans and mice, and their expression is regulated by gut microbiota and can promote insulin resistance in WAT [18]. Additionally, there may be a gut–microbiota–miRNA-122 connection that instigates obesity-associated insulin resistance in C57BL/6 mice [82]. For example, the circulatory and hepatic miRNA-122 family causes decreased PPAR-α expression in WAT, resulting in an increased release of free fatty acids to the circulation and consequent WAT and liver inflammation and insulin resistance in mice models [61,113]. *Bacteroides uniformis* (*Bacteroidetes phyla*) is identified as a regulator of miRNA-122-5p in human subjects that targets forkhead box O (FoxO), which is linked with increased gluconeogenesis-related genes and can also be stimulated via PPARγ to modulate glucose metabolism [87,114]. Furthermore, an increase in the abundance of *B. acidifaciens* (*Bacteroidetes phyla*) is linked with reduced insulin resistance in C57BL/6 mice [81,115].

Obesity-associated inflammation contributes to insulin signaling impairment through numerous circulating cytokines and also by directly impacting cytokine-producing organs, such as the liver and muscle tissue [98]. For example, in vitro studies of 3T3-L1 adipocytes have shown that TNF-α inhibits insulin receptor activity by reducing PPAR-γ expression [98]. While several miRNAs are altered with obesity [62,116], Assmann et al. showed that circulatory miRNA-15a-5p was negatively related to *H. parainfluenza* (*Proteobacteria phyla*) and insulin level in human subjects [86]. miRNA-15a-5p inhibits the gene expression of uncoupling protein (UCP) 2, leading to increased ATP levels and improved glucose-dependent insulin secretion in the pancreatic beta cells of human subjects [86,117]. Lastly, it was shown in a mice study that the higher abundance of *Bacteroides* (*Bacteroidetes phyla*), *Parabacteroides* (*Bacteroidetes phyla*), and *Bilophila* (*Proteobacteria phyla*) is positively correlated with hepatic miRNA-5112, which regulates genes that could contribute to insulin resistance, such as *Akt1*, *Socs3*, potassium calcium-activated channel subfamily N member 1(*Kcnn1)*, L-selectin mediated binding receptor domain containing 1(*Lmbrd1)*, and growth factor receptor-bound protein 10 (*Grb10)* [89].

Oxidative stress, the imbalance between antioxidative defense and reactive oxygen species (ROS) generation [118,119], also prepares the microenvironment for the propagation of inflammation and the subsequent obese phenotype [118,119]. *D. longicatena* (*Proteobacteria phyla*) is associated with increased expression of circulating miRNA-185-5p, which is related to decreased expression of glutathione peroxidase 1 (GPx1), an important antioxidant enzyme for catalyzing and detoxifying H2O2 in human endothelial cells [120]. Thus, the *D. longicatena*–miRNA-185-5p connection might be considered in the context of reduced antioxidant defense and increased oxidative stress, DNA damage, and the increased biosynthesis of cholesterol, thereby facilitating the progression of obesity-associated metabolic disorders [120,121,122].

Gut microbiota metabolites, such as SCFAs, are important mediators of microbiota–cell interactions and immunomodulation and generally link host nutritional status to intestinal homeostasis [123]. Importantly, treatment with SCFA in mice decreases LPS-induced inflammation in the intestines, which is mediated by increased miRNA-145, which subsequently decreases dual-specificity phosphatase 6 (DUSP6), an enzyme that regulates the MAPK signaling pathway involved in inflammatory response, therefore highlighting a potential therapeutic effect for controlling intestinal inflammation [124,125]. Using fecal material transfer methods, Du et al. reported that an increase in SCFA-producing bacteria (specifically acetate and butyrate) as a result of microbiota transplantation affects the WAT miRNA-378a family and consequently decreases expression of the Yin Yang 1 (*YY1*) gene that is associated with improved lipid and glucose metabolism in Kunming mice [83].

A limitation of this review that must be acknowledged is the low number of studies with human subjects, which included only two studies. Additionally, the reviewed animal studies recruited different strains and sexes of mice, including male C57BL/6J and female Kunming mice, and different durations of dietary intervention. Additionally, although several of the included studies established clear statistical associations between miRNA profiles and gut microbiota, others did not include such analyses, which can limit the ability to make strong comparisons across the findings. Furthermore, although remarkable correlations have been observed between gut microbiota and miRNA expression, it is important to acknowledge that these relationships are only associative. While the bidirectional interactions between the microbiome and miRNA can be linked to promising insights into the pathophysiology of obesity, functional validation by the use of well-designed studies is necessary to elucidate mechanistic aspects and confirm their roles in the development or progression of obesity and its comorbidities.

Future research should include utilizing FMT in the context of obesity to elucidate specific connections and interactions between gut microbiota composition and miRNA expression and related mechanisms [126]. The interplay between miRNAs and gut microbiota is a dynamic and multifaceted relationship, since diet is the primary determinant of the gut microbiota profile and shifts in gut microbiota triggered by specific macronutrients can modulate host miRNA expression, creating a bidirectional communication loop [127,128,129,130]. Since diet is a primary contributor to the gut microbiome, a large gap exists for determining the gut microbiota–miRNA mechanisms of bioactive nutrients with anti-obesity potential. For example, supplementation with plant extracts in high-fat fed C57BL/6J mice restored dysbiosis and was associated with increased miRNA-122, which is normally downregulated in obesity and liver disease [82]. Du et al. also showed that betaine supplementation can ameliorate high-fat diet-induced gut microbiota dysbiosis and increase SCFA-producing bacteria. which in turn can affect miRNA-378a and modulate lipid metabolism [83]. These examples highlight the likely contribution of gut microbiota–miRNA connections as a mechanisms of bioactive nutrients.

Additionally, gut microbiota–miRNA connections should be evaluated in the pathology of colorectal cancer, since obesity-associated dysbiosis aids in the development of the disease and incidence is increasing among younger individuals [131]. One example that can be mentioned is miRNA-21, which is associated with colorectal cancer progression and is elevated with hepatocyte insulin resistance and steaosis [132,133]. Furthermore, Blasco-Baque et al. reported a significant positive correlation between hepatic miRNA-21 and *B. acidifaciens*, alongside a significant negative correlation between hepatic miRNA-21 and *Firmicutes* [81]. The diversity of the gut microbiome strengthens the response to traditional colorectal cancer therapies, highlighting a potential role for the utilization of FMT as a personalized treatment plan for those with obesity-associated colorectal cancer [134,135,136].

Currently, there is a very high demand for the new anti-obesity, glucagon-like peptide1 (GLP1) agonists medications [137]. The GLP1 agonists, specifically Liraglutide, impact gut microbiota composition in diet-induced obese mice models and alter *Verrucomicrobiota* and *Proteobacteria* populations [138]. These medications are also associated with changes in the expression of different miRNAs such as miRNA-34a, miRNA- 21, miRNA-132, and several others [139]. Therefore, assessing GLP-1 agonists/miRNA–microbiota interactions in the setting of obesity pathophysiology may provide innovative approaches to understanding variability in treating obesity and its comorbidities.

## 5. Conclusions

**Figure 3:**
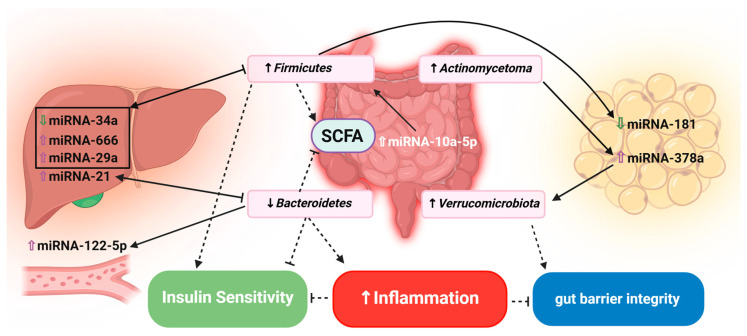
A complex interplay between gut microbiota and miRNA exists in relation to obesity pathophysiology and impacts several important metabolic parameters, including insulin signaling and inflammation. (Dashed arrows represent mechanistic connection; capped arrows indicate the counter direction of arrow shown for bacteria.) [16,77,78,82,84,85,86].

In conclusion, there are numerous gut microbiota–miRNA bidirectional interactions and associations contributing to obesity pathophysiology that involve WAT, the liver, and the GI tract and impact pathways such as inflammation, oxidative stress, gut barrier integrity, and adipogenesis. *Firmicutes*, *Bacteroidetes*, and *Akkermansia* (*Verrucomicrobiota phyla*) among bacteria, and the miRNA-34a, miRNA-181, and miRNA-378a family among the miRNAs, play key roles in this context. Figure 3 summarizes the gut microbiota–miRNA interplay and the involved organs and pathways.

There are diverse gut microbiota and miRNA interactions in obesity, but the exact pairings between bacteria and miRNA varies depending on experimental conditions, including tissue type, diet, host genetics, and the microbiota or miRNA that are examined [140]. This bidirectional interaction highlights new research opportunities in the context of obesity pathophysiology that may lead to the development of new therapeutic targets for obesity and its comorbidities.

## Figures and Tables

**Table 1:** Key characteristics and associations identified in the included animal studies.

Animal Strain	N (Sex)	Diet/Control Diet	Duration (Weeks)	Impact on Gut Microbial Community		Impact on miRNA			Ref.
				Increased	Decreased	Increased	Decreased	Site	
C57BL/6	20 (M)	HFD/NCD	6		*Firmicutes* (P) *Clostridium* (G)	miRNA-181		WAT	Virtue et al., 2019 [18]
C57BL/6	62 (M)	HFD/NCD	12	*Firmicutes* (P) * Oscillopsia (G) *Clostridium* (G)	*Bacteroidetes* (P) * B. *acidifies*		miRNA-21 * miRNA-666 miRNA-181	Liver	Blasco-Baque et al., 2017 [81]
C57BL/6	(M)	HFD/SD	32	*Firmicutes* (P) *Lactobacillus* (G) *Ruminiclostridium* (G) *Lachnoclostridium* (G)		miRNA-29a		Liver	Yang et al., 2023 [85]
C57BL/6J	24 (M)	HFD/SD	24	*Bacteroidetes* (P)	*Firmicutes* (P)	miRNA-122	miRNA-34a	Liver	Cossiga et al., 2021 [82]
Kunming mice	24 (F)	NCD/HFD	23	*Verrucomicrobia* (P) *Akkermansia* (G), *Firmicutes* (P) *Lactobacillus* (G) *Actinomycetoma* (P) *Bifidobacterium* (G)		miR-378a-3p/5p		WAT	Du et al., 2021 [83]
C57BL/6J	126 (M)	HFD/LFD	8	*Actinomycetoma* (P) *Lachnospiraceae* (F)		miRNA-10a-5p		Orally administered	Guo et al., 2020 [84]

Table notes: HFD: High Fat Diet; NCD: Normal Chow Diet (balanced low-fat diet commonly used as a control in metabolic studies. Contains 5% fat); SD: Standard Diet (standard rodent diet with a complete nutritional profile, adjusted according to research needs, contains 3% fat); LFD: Low Fat Diet (specifically reduced fat content); P: Phyla; G: Genus; WAT: White Adipose Tissue; * indicates significant correlation, *p* < 0.05.

**Table 2:** Key characteristics and associations identified in the included human studies.

N	Age	Gender	BMI (kg/m^2^)	Characteristics	Impact on Gut Microbiota Community		Impact on miRNA			Ref.
					Increased	Decreased	Increased	Decreased	Site	
103	Cases: 46.6 ± 9.4 Controls: 44.7 ± 9.1	Cases: 28 (M), 50 (F) Controls: 10 (M), 15 (F)	Cases: 30–40 Controls ≤ 25	Cases with obesity	*Bacteroidetes* (P) *B*. *eggerthi* (G) * H. *parainfluenzae* (G) *	-	miRNA-375 and miRNA-183-5p *	miRNA-15a-5p *	serum	Assmann et al., 2020 [86]
60	Cases: 48.13 ± 6.56 Controls: 46.27 ± 8.44	Cases: 14 (M), 16 (F) Controls: 13 (M), 17 (F)	Cases: 23.70 ± 3.00 Controls: 22.37 ± 1.87	Cases with diabetes mellitus	-	*Bacteroidetes* (P) *Bacteroides*. *Uniformis* (G) * Firmicutes (P) *Phascolarctobacterium* *Faecium* (G) *	miRNA-122-5p *		serum	Li et al., 2020 [87]

Table notes: P: Phyla; G: Genus; * indicates significant correlation, *p* < 0.05.

## Data Availability

Data are contained within the article.
